# The United States Medical Licensing Exam Step 2 Clinical Skills Examination: Potential Alternatives During and After the COVID-19 Pandemic

**DOI:** 10.2196/25903

**Published:** 2021-04-30

**Authors:** Rawish Fatima, Ahmad R Assaly, Muhammad Aziz, Mohamad Moussa, Ragheb Assaly

**Affiliations:** 1 Department of Internal Medicine University of Toledo Medical Center Toledo, OH United States; 2 College of Medicine and Life Sciences The University of Toledo Toledo, OH United States; 3 Division of Gastroenterology and Hepatology University of Toledo Medical Center Toledo, OH United States; 4 Department of Emergency Medicine University of Toledo Medical Center Toledo, OH United States; 5 Division of Pulmonary and Critical Care Medicine University of Toledo Medical Center Toledo, OH United States

**Keywords:** USMLE, United States Medical Licensing Examination, The National Resident Matching Program, NRMP, Step 2 Clinical Skills, Step 2 CS, medical school, medical education, test, medical student, United States, online learning, exam, alternative, model, COVID-19

## Abstract

We feel that the current COVID-19 crisis has created great uncertainty and anxiety among medical students. With medical school classes initially being conducted on the web and the approaching season of “the Match” (a uniform system by which residency candidates and residency programs in the United States simultaneously “match” with the aid of a computer algorithm to fill first-year and second-year postgraduate training positions accredited by the Accreditation Council for Graduate Medical Education), the situation did not seem to be improving. The National Resident Matching Program made an official announcement on May 26, 2020, that candidates would not be required to take or pass the United States Medical Licensing Examination Step 2 Clinical Skills (CS) examination to participate in the Match. On January 26, 2021, formal discontinuation of Step 2 CS was announced; for this reason, we have provided our perspective of possible alternative solutions to the Step 2 CS examination. A successful alternative model can be implemented in future residency match seasons as well.

COVID-19, a novel disease caused by SARS-CoV-2, was first recognized in Wuhan, China, in late 2019; it continued to spread globally, leading to a pandemic [1]. Efforts are being implemented to control this pandemic, prevent health care services from being overwhelmed, and minimize the effects of the pandemic on the economy while work progresses on vaccine development and antiviral therapy. The surging demands on medical systems have forced hospitals to make modifications such as deploying specialists in intensive care units and emergency departments and inviting medical students to graduate early and start working as interns.

The National Resident Matching Program (NRMP) residency match (“the Match”) was also affected. Recommendations regarding limited travel and continued social distancing for the health and safety of applicants and program staff were taken into consideration. Adding to the uncertainty, on May 26, 2020, NRMP announced suspension of the United States Medical Licensing Examination (USMLE) Step 2 Clinical Skills (CS) examination for a period of 12-18 months. It was stated that “The NRMP does not specifically require applicants to take or pass the CS examination in order to participate in the Match. The NRMP requires that US applicants meet the requirements for graduation set by their medical school and the eligibility criteria set by their matched residency training program. International medical graduate (IMG) applicants must meet the exam requirements set by the Educational Commission for Foreign Medical Graduates (ECFMG) to achieve ECFMG certification [2].” ECFMG later announced that they would accept the Occupational English Test for health care. Listening, Reading, Writing, and Speaking are the components that are tested in this examination [3]. Remote proctoring was established to provide wide availability for applicants. On January 26, 2021, formal discontinuation of Step 2 CS was announced [4]. The eligibility criteria for taking the Step 3 examination were modified, and completion of Step 2 CS was no longer required to take the Step 3 examination. ECFMG introduced pathways for IMGs to obtain ECFMG certification.

The first round of clinical skills testing for all medical students under the name of Step 2 CS was conducted by USMLE in 2004 at a national level. Before 2004, an analogous exam, the Clinical Skills Assessment, was used to assess the clinical skills of foreign medical graduates [5]. The Step 2 CS exam was conducted by the Clinical Skills Evaluation Collaboration at six test centers (Atlanta, Chicago, Illinois, Houston, Los Angeles, and Philadelphia) within the United States. The state medical licensing boards delineated that the aim of this examination was “to ensure the ability to communicate effectively with patients and colleagues along with standards of safe practice of medicine.” The examination had three components: Communication and Interpersonal Skills (CIS), Spoken English Proficiency (SEP), and Integrated Clinical Encounter (ICE). During this examination, examinees encountered 12 standardized patients and were given 15 minutes to take a complete history and perform a clinical examination for each patient; they were then given 10 additional minutes to write a patient note describing the findings and to generate an initial differential diagnosis list and a list of initial tests. The objectives of this examination were to assess communication skills, collect and provide information, assist patients with decision-making, provide emotional support to patients, gather data, and assess English language proficiency [6].

In a study published by Rosenthal et al in 2019 [7], an analysis was performed of 1041 graduates of a medical school from 2014-2017. The authors observed that candidates who failed the Step 2 CS examination had risk factors such as low National Board of Medical Examiners scores, low Objective Structured Clinical Examination (OSCE) scores, and poor faculty ratings. Thus, one can presume a direct correlation between the Step 2 CS examination performance of global applicants and their performance on other standardized examinations. Mehta et al [8] expressed their views in an article published in 2005, titled “A Critique of the USMLE Clinical Skills Examination,” in which the authors expressed frustration regarding unhelpful feedback from their Step 2 CS score reports as compared to other USMLE examinations.

As with everything else that has been changing in medical education in the last few months, it is worth visiting the question of whether the Step 2 CS examination needs to change. The expense and travel involved do not currently seem to be very practical, which leads to the idea of administering a gateway virtual assessment instead. Consideration should be given to the cost of the examination (US $1600), time and money spent on traveling, date availability in limited centers, and visa issues being faced by IMGs, while simultaneously considering the need for an alternate standardized performance assessment of US and international candidates. The aforementioned challenges are not concealed; in fact, the often-used guide, *First Aid for the USMLE Step 2 CS* [9], offers pages of lists of transportation, restaurants, and hotels with varying price points in these major cities to attempt to alleviate stressors for candidates.

The nonuniformity of OSCE and examination patterns in international medical schools raises the question of possible solutions to prevent non-US physicians from demonstrating subpar performance. The USMLE Step 2 CS website reports a pass rate of 94% (ICE 96%, CIS 98%, SEP >99%) for candidates from US and Canadian medical schools on the first attempt and 73% (ICE 81%, CIS 94%, SEP 93%) for candidates from non-US/Canadian schools [10]. These statistics are reflective of the continuing need to practice prerequisite assessments before granting an interview at the minimum for IMGs.

In 2016, the Association of American Medical Colleges (AAMC) launched an initial pilot program of standardized video interviews (SVIs) for all emergency medicine residency applicants; however, AAMC decided that there would be no SVIs beginning in the 2020-21 residency application cycle. The purpose of these interviews was to assess an applicant’s “Knowledge of Professional Behaviors and Interpersonal and Communications Skills.” Although it was stated that the AAMC reckoned the SVI to be a reliable and valid assessment, the decision to not expand the SVI to other residencies and to discontinue its use in emergency medicine was due to lack of and sometimes hesitant use of SVI in the selection process [11]. We believe that the most important part of the examination is demonstrating the ability to communicate with a patient. A study published in 2014 showed that communication issues were often the top reason for complaints against physicians in North Carolina [12]. Another study showed a modest correlation between Step 2 CS Communication and Interpersonal Skills ratings and the communication skills of interns [13].

Given the need of the hour, it may be the right time to revisit the idea of the SVI. A new version of the SVI can be conducted with two components: clinical and communication examinations (Figure 1). The communication part can be conducted at any place and time. Candidates will need to record their responses to the questions sent to them via a single-use web link with a time limit provided by USMLE and will be required to send the responses back for evaluation. The purpose of this examination will be to assess interpersonal communication and decision-making skills. For the assessment of clinical skills and history taking, Prometric staff can be trained in different countries to simulate patients, and the recorded encounters can then be sent to the examiners to assess and score. This step will not only help with the cost of the examination but will also decrease the stress of travelling and scheduling for all candidates, including national and international candidates.

**Figure 1:**
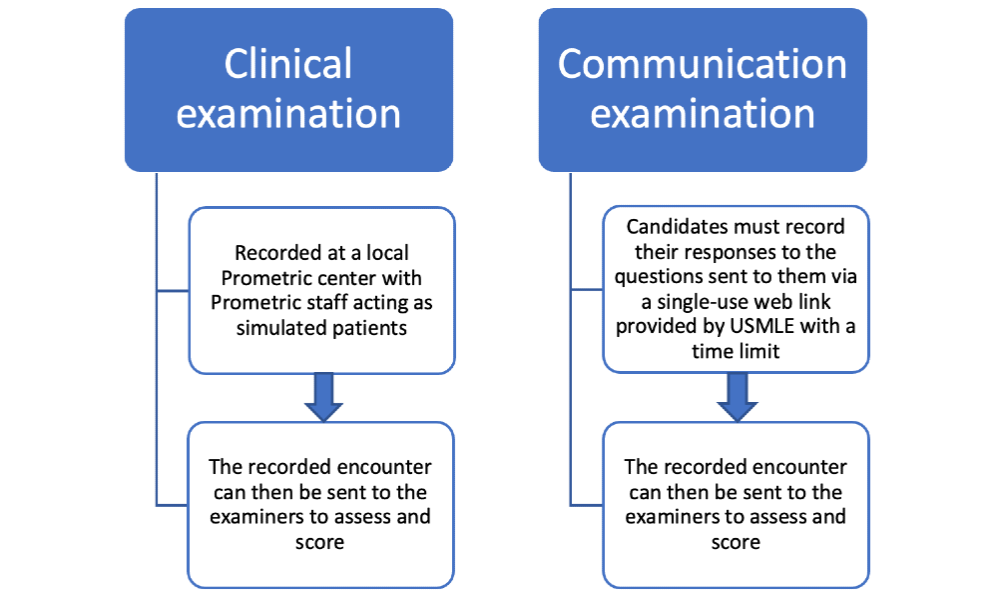
Pictorial illustration of the standardized video interview model. USMLE: United States Medical Licensing Examination.

Other alternate solutions could be to provide training in these clinical and soft skills during the first 6 months of intern year or relying on the candidate’s performance on medical school and other USMLE exams. This approach may result in more focus on OSCE examinations during medical school training. A study published in 2015 [14] showed that US medical students did not perform well on physical examinations, especially musculoskeletal and neurology examinations. Further examining student performance and having medical schools focus on their weaknesses may eradicate the need to conduct Step 2 CS for American medical graduates. Most medical students at the University of Toledo Medical Center expressed that they felt more than prepared for their physical skills examinations because of the multidisciplinary approach taken at their school. They discerned that as they needed to fit the scheduling and cost of this examination into their busy fourth year schedule, the experience was not worthwhile. They stated that they do not believe it is necessary to test their proficiency in speaking to patients again, as this proficiency is tested and improved upon each day on the wards.

Moving toward a virtual examination based on the model of SVI, relying on medical school examination performance, and provision and grooming of skills during internship instead of conducting USMLE Step 2 CS are some adaptations that seem like they can be given consideration. Well-designed and conducted studies are needed to provide further information and may lead to dramatic changes in the testing and interview process.

## Abbreviations

AAMC: Association of American Medical Colleges
CIS: Communication and Interpersonal Skills
CS: clinical skills
ECFMG: Educational Commission for Foreign Medical Graduates
ICE: Integrated Clinical Encounter
IMG: international medical graduate
OSCE: Objective Structured Clinical Examination
SEP: Spoken English Proficiency
SVI: standardized video interview
USMLE: United States Medical Licensing Examination
